# Time to initiation of antenatal care and its predictors among pregnant women across 35 sub-Saharan African countries (2011–2024): A multilevel mixed-effects acceleration failure time model of multination population survey data

**DOI:** 10.1186/s12978-026-02335-8

**Published:** 2026-04-23

**Authors:** Abdulkerim Hassen Moloro, Oumer Abdulkadir Ebrahim

**Affiliations:** 1 Department of Nursing, College of Medicine and Health Sciences, Samara University, Samara, Ethiopia; 2 Department of Public Health, College of Medicine and Health Sciences, Samara University, Semera, Ethiopia

**Keywords:** Antenatal care, Demographic Health Survey, Sub-Saharan Africa, Timely, Women

## Abstract

**Background:**

Despite the well-established link between delayed initiation of antenatal care (ANC) and negative pregnancy and childbirth outcomes, a substantial proportion of women in sub-Saharan Africa continue to begin ANC late in their pregnancies. The previous studies failing to incorporate the most current information, confined to a single country or small number of nations and employed statistically flawed proportional hazard models with violated assumptions compromising the reliability of the conclusions. Therefore, this study aimed to investigate the timely initiation of ANC and its determinants among pregnant women in 35 Sub-Saharan African countries by utilizing mixed-effects acceleration failure time model based on the most recent (2011–2024) Demographic and Health Survey data.

**Methods:**

A retrospective follow-up study using secondary data from the Demographic and Health Surveys of 35 sub-Saharan African countries conducted between 2011 and 2024. A total weighted sample of 222, 866 women aged 15–49 years who had Antenatal care visits during their current or most recent pregnancy within the 5 years prior to the survey were included in the analysis. Descriptive and inferential analyses were performed using STATA 17. A multistage stratified cluster sampling technique was employed. The Kaplan-Meier (KM) method was used to estimate time to first antenatal care visit. A multilevel mixed-effects Weibull acceleration failure time survival model was applied to determine predictors. Model adequacy was assessed by using the proportional hazard assumption using scaled Schoenfeld residuals. The adjusted acceleration factor with 95% confidence intervals were used to indicate statistical significance and the strength of associations. Model comparison was evaluated by model statistical summary such as Akaike’s Information Criterion (AIC), Bayesian Information Criterion (BIC), log-likelihood (LL), and deviance (-2*LL).

**Result:**

In this study, the estimated mean survival time for pregnant women to initiate their first ANC visit across 35 sub-Saharan African countries was 6.9 months (95% CI: 6.89–6.92). Women age 15–24 (AAF = 0.88; 95% CI: 0.86, 0.90), being rural residence (AAF = 0.91; 95% CI: 0.90, 0.93), women with no formal, primary, and secondary education respectively (AAF = 0.95, 95% CI: 0.92, 0.98; AAF = 0.84, 95% CI: 0.82, 0.87; AAF = 0.83, 95% CI: 0.80, 0.85), home delivery (AAF = 0.82; 95% CI: 0.81, 0.83), single marital status (AAF = 0.96; 95% CI: 0.95, 0.98), being not exposed to mass media (AAF = 0.90; 95% CI: 0.88, 0.91), poor and middle wealth index respectively (AAF = 0.96; 95% CI: 0.94, 0.98; AAF = 0.92; 95% CI: 0.90, 0.94), multiparous (AAF = 0.91; 95% CI: 0.89, 0.93), grand multiparous (AAF = 0.79; 95% CI: 0.77, 0.80), and low community Antenatal care utilization (AAF = 0.39; 95% CI: 0.38, 0.40) were predictors of time to first ANC visit.

**Conclusion:**

The timely initiation of the antenatal care (ANC) in the first trimester remains a significant challenge across 35 sub-Saharan African countries, with a prevalence of only 41.5% and a mean delay of nearly seven months before the first visit. This low rate was influenced by sociodemographic, obstetrics and community level factors including young maternal age, rural residence, home delivery, lower educational status and wealth index, multi and grand multiparous, being not exposure to mass media and low community ANC utilization. To address this gap, community-level interventions such as mobilizing peer leaders in low-utilization areas may be as critical as individual-level efforts like financial support for transport and adolescent-friendly services. Integrating ANC promotion with existing media campaigns and reproductive health program could efficiently shift norms and improve early uptake. Future research must incorporate health system and country level variables and employ longitudinal designs to establish casualty and inform more effective policies aimed at making timely ANC initiation a cornerstone of maternal health in the region.

**Supplementary Information:**

The online version contains supplementary material available at 10.1186/s12978-026-02335-8.

## Introduction

Antenatal care (ANC) is a vital component of the maternal and child health continuum, designed to ensure the well-being of both mother and fetus throughout pregnancy [1]. It encompasses early detection, prevention, and management of pregnancy-related complications, alongside health education and promotion [2]. According to the World Health Organization’s Focused Antenatal Care model, it is recommended that all pregnant women initiate ANC within the first 12 weeks of gestation to optimize outcomes [3]. However, global data indicates that only 43% of women begin ANC during this critical period, with stark regional disparities: approximately 85% in developed countries, under 45% in developing regions, and less than 25% in sub-Saharan Africa [4]. In Ethiopia specifically, just 42.7% of pregnant women attend their first ANC visit during the first trimester, highlighting a significant gap in timely maternal health service utilization [5].

A range of factors have been found to contribute to the timely initiation of antenatal care (ANC). Key sociodemographic determinants include maternal age [6], place of residence [7, 8], marital status [7, 9], level of education [10, 11], occupation [12], household wealth index [7, 8], family size [8], and partner cohabitation. Additionally, logistical and systemic barriers such as long distances to health facilities [6, 12, 13] and lack of health insurance coverage [14] have been associated with late ANC attendance. Beyond these, reproductive and behavioral factors also play a role such as higher birth order [7, 8, 15], unplanned pregnancies [9, 11], absence of prior institutional deliveries [13], limited experience with previous ANC services [9], poor understanding of ANC benefits [10], and misconceptions about the appropriate timing for the first visit [9]. Together, these factors highlight the complex interplay of personal, social, and structural influences that hinder timely access to maternal healthcare.

Globally, a range of initiatives have been implemented to enhance maternal and newborn health, with a particular focus on addressing the challenge of delayed antenatal care (ANC) initiation [16]. A key milestone was the World Health Organization’s introduction of a revised ANC model in 2016, which increased the recommended minimum number of ANC contacts from four to eight. This shift was designed to create more opportunities for early identification and management of pregnancy-related health risks. In addition to this, both health system and community-level interventions have been deployed to strengthen service quality and accessibility, while also raising public awareness about the importance of timely ANC engagement [6, 17–19]. These efforts have been especially concentrated in resource-constrained settings, where barriers to early care are most pronounced [19, 20].

Despite the well-established link between delayed initiation of antenatal care (ANC) and negative pregnancy and childbirth outcomes [21–24], a substantial proportion of women in resource-constrained regions such as Africa continue to begin ANC late in their pregnancies. Existing research on the timing of the antenatal care (ANC) initiation in Sub-Saharan Africa (SSA) has primarily been limited to single country analysis or small multi-country studies, which restricts the generalizability of findings and the ability to draw comparative insights across diverse socio-economic and health care context [8, 10, 11, 13, 15].

While studies such as Oyato et al.(2024) in Ethiopia [5] and Alem et al.(2022) [25] across several SSA countries have identified key individual and community level factors, these analysis often rely on older Demographic and Health Survey(DHS) rounds and do not incorporate the most recent data spanning up to 2024. Moreover, many studies use proportional hazards models despite frequent violations of the proportional hazard’s assumptions, limiting the robustness of inferences [26]. In contrast, this study pools nationally representatives DHS datasets from 35 sub-Saharan African countries conducted between 2011 and 2024, thereby capturing both common regional determinants and country specific variations. By employing a multilevel mixed-effects Weibull accelerated failure time (AFT) model, this study addresses methodological shortcomings of earlier work and provide interpretable results in terms of delays in months rather than abstract hazard ratios. Therefore, it’s important for a comprehensive, up-to-date, and methodological advanced multi-country analysis that utilizes recent DHS data from 35 SSA countries nations to provide a pan regional perspective on both the trends and determinants of ANC initiating timing.

## Methods

### Study area, design, period and data source

This study conducted secondary analysis using data from the Demographic and Health Surveys (DHS) of 35 countries in Sub-Saharan Africa (SSA). The countries involved were Angola, Burkina Faso, Benin, Burundi, DR Congo, Republic of the Congo, Côte d’Ivoire, Cameroon, Ethiopia, Gabon, Ghana, Gambia, Guinea, Kenya, Comoros, Liberia, Lesotho, Madagascar, Mali, Mauritania, Malawi, Mozambique, Nigeria, Niger, Namibia, Rwanda, Sierra Leone, Senegal, Chad, Togo, Tanzania, Uganda, South Africa, Zambia, and Zimbabwe. The selection of country was based on the recent survey year (2011–2024), availability of a standardized and unrestricted dataset, and presence of observations for the outcome variable in the datasets.

The Demographic and Health Surveys (DHS) across all countries employed a cross-sectional design to collect data on sociodemographic characteristics and key health indicators, including maternal health. For this analysis, we included countries with recent DHS rounds conducted between 2011 and 2024. The data analyzed in this study were derived from the DHS under-5 children’s file (KR) and individual women’s files across 35 countries.

While single-country analyses provide valuable insights, they often suffer from limited sample sizes, contextual biases, and reduced external validity. By pooling nationally representative DHS datasets across multiple countries, this study captures the heterogeneity of maternal health behaviors and system-level influences across diverse socio-economic, cultural, and geographic contexts. This approach strengthens statistical power, improves precision of estimates, and allows identification of both common regional determinants and country-specific variations in antenatal care initiation. Consequently, pooling provides a more comprehensive and policy-relevant understanding of maternal health challenges in Sub-Saharan Africa.

### Population and sampling technique

The source population for this study comprised all reproductive age women in 35 SSA countries. The study population included all pregnant women aged 15–49 years who had ANC during their current or most recent pregnancy within the 5 years prior to the survey were included in this study. Across all countries, the surveys used a multistage stratified cluster sampling technique to select the study participants. In the first stage, each country was divided into clusters, and clusters were randomly selected based on the probability proportional to their contribution to overall country’s population. In the second stage, using the housing census as a sampling frame, a representative number of households was selected from each cluster.

This study pooled DHS data from 35 countries (2011–2024) to maximize sample size, enhance statistical power, and capture regional heterogeneity. Finally, after handling the missing observations, a weighted sample of 222, 866 women aged 15 to 49 years with complete data on the variables of interest were included in the study (Table 1). Furthermore, the DHS employs nationally representative sampling designs and standardized data collection across countries, pooling data from 35 diverse sub‑Saharan African nations enhances the external validity of our findings. This approach ensures that the results are broadly representative of pregnant women across the region’s varied socio‑economic and health system contexts. Details about DHS methodology can be accessed online (https://dhsprogram.com/Methodology/index.cfm).

**Table 1:** Survey years and sample sizes of women aged 15–49 years from 35 Sub-Saharan African (SSA) countries included in the study, from 2011 to 2024

Country	Survey year	Unweighted sample size	Weighted sample size
Angola	2015-2016	7041	6960
Burkina Faso	2021	6376	6326
Benin	2017-2018	7964	8045
Burundi	2016-2017	8600	8879
DR Congo	2013-2014	9711	9276
Congo	2011-2012	5879	5478
Cote d’Ivoire	2021	5330	5062
Cameroon	2018-2019	5641	5764
Ethiopia	2019	2935	2923
Gabon	2019-2021	4231	4252
Ghana	2022-2023	5083	4614
Gambia	2019-2020	5771	5344
Guinea	2018	4733	4719
Kenya	2022	10014	9286
Comoros	2012	1875	1921
Liberia	2019-2020	4177	3957
Lesotho	2023-2024	1471	1364
Madagascar	2021	8236	8277
Mali	2018	4936	5347
Mauritania	2019-2021	6793	6831
Malawi	2015-2016	13217	13270
Mozambique	2022-2023	4706	4726
Nigeria	2018	16427	16575
Niger	2012	6507	6851
Namibia	2013	3824	3716
Rwanda	2019-2020	6039	6159
Sierra Leone	2019	7276	7213
Senegal	2023	5456	5155
Chad	2014-2015	6615	7176
Togo	2013-2014	4655	4504
Tanzania	2022	5214	5292
Uganda	2016	10074	9957
South Africa	2016	5768	5725
Zambia	2018-2019	7283	7244
Zimbabwe	2015	4570	4665
Total		**224, 387**	**222, 866**

### Variable selection and data construction

While the Demographic and Health Surveys (DHS) employ standardized data collection tools, sampling procedure to ensure comparability across countries and face‑to‑face interviews to ensure comparability across countries, there are important differences in timing and survey segments. Each country conducts DHS at different intervals, and while the core maternal health modules are consistent, certain contextual variables may vary or excluded. To address this, we harmonized variable across datasets using the Guide to DHS Statistics and applied survey weight to maintain representatives.

For this study, we focused on pregnant women by drawing data from two primary DHS file types: the individual women’s questionnaire (IR file) and the children’s file (KR file), which contains reproductive and maternity histories for births within the five years preceding each survey. Women with missing information on antenatal care timing were excluded in accordance with DHS guidelines (Guide to DHS Statistics, DHS‑8) [27].

Independent variables were selected based on theoretical relevance to antenatal care initiation as established in previous literature [5, 28], consistent availability across all included DHS surveys; and statistical considerations, including significance in preliminary analyses and absence of multicollinearity. To construct the analytic dataset, we identified all Sub‑Saharan African countries with DHS surveys conducted between 2011 and 2024 that contained complete data on antenatal care timing. The IR and KR files were merged using unique identifiers to link each woman to her most recent pregnancy. Country‑specific datasets were then appended into a single pooled dataset, retaining country identifiers and survey weights (v005) to account for the complex stratified cluster sampling design. All variables were harmonized and recoded following the Guide to DHS Statistics to ensure comparability across surveys.

### Dependent variable

#### Time to initiate antenatal care

The time in months that pregnant women take to receive the first ANC service is the dependent variable. The event of interest considered as success (event = 1) is if women had ANC in the first trimester of pregnancy and otherwise censored (censored = 0) [5].

####  Independent variables

**Fig. 1:**
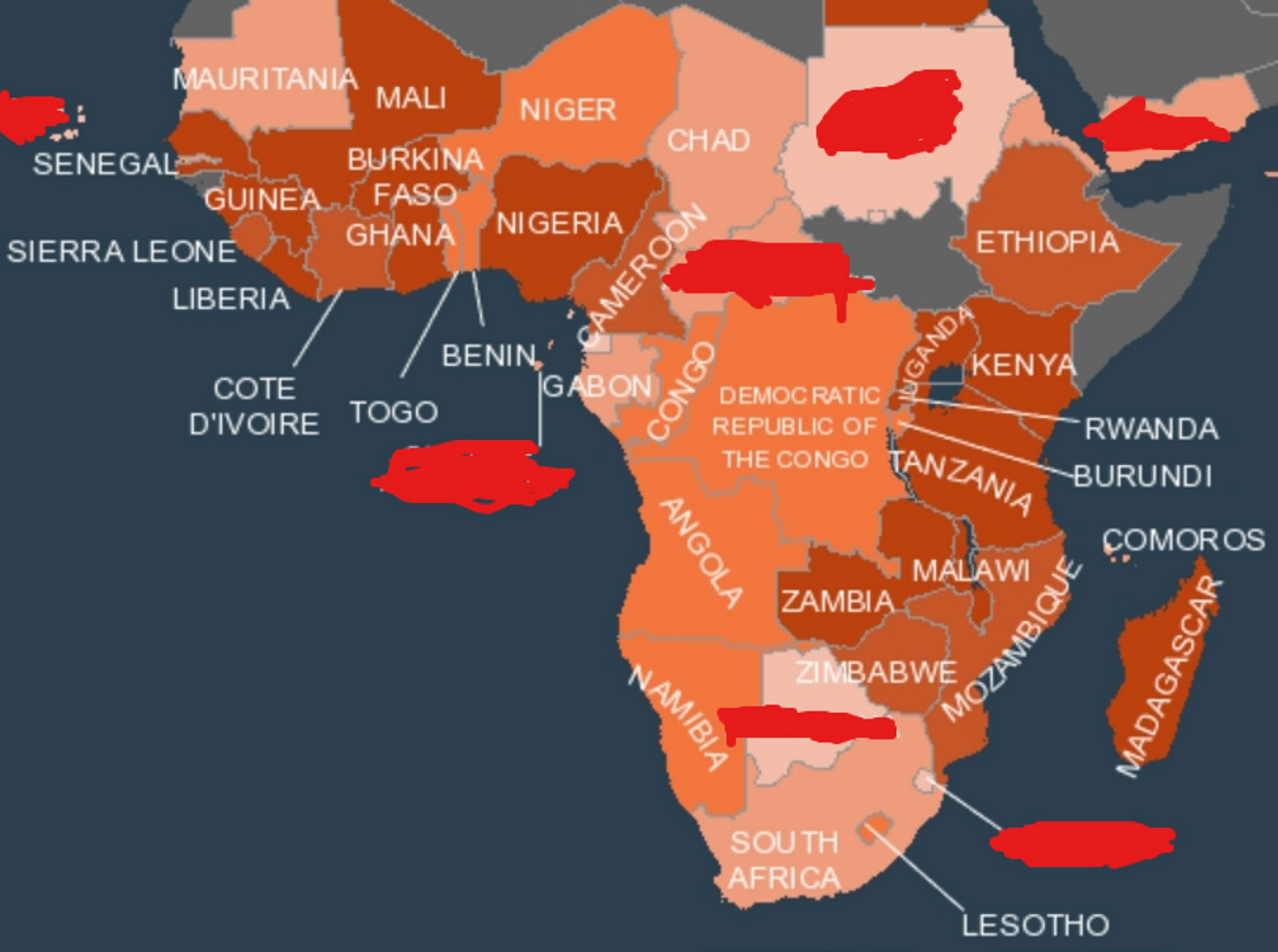
Map showing the 35 Sub‑Saharan African countries included in the study, grouped into West, East, Central, and Southern Africa. (Source: DHS Program)

In this study, a total of 19 independent variables were included, selected based on their availability in the DHS dataset and prior evidence of association with women’s antenatal care (ANC) follow-up [5, 28]. These variables were grouped into three categories: sociodemographic and economic factors, obstetric factors, and community-level factors. *Sociodemographic and economic variables* encompassed place of residence (urban or rural), geographical sub-region countries (West, East, Central, Southern) (Fig. 1), educational levels (no education, primary, secondary, and higher), maternal age, marital status, household size(1–4, 5–7 and ≥ 8), exposure to media (exposed to at least one of radio, magazine/newspaper or television were labeled as ‘yes’ and those who did not were labeled as ‘no’), place of delivery(home/health facility), distance to health facility (big problem/not a big problem), and household wealth index(poor, middle and rich). *Obstetric factors* included birth interval, parity, contraception method used, postnatal check within two months, cesarean section in the last birth, and history of child loss.

*Community level variable* included; *community women’s education* (measured as the proportion of women who completed primary and above educational level in the primary sampling unit. It was categorized as low and high if less than median and more than median of study population of the cluster had at least eight years of education respectively), *community ANC utilization* (proportion of the mothers who had had prenatal care at least four times ANC utilization), and *community-level poverty* (computed from the household wealth and defined as the proportion of women in the top 3 wealth quantiles in the clusters. It was categorized as low and high if less than median and more than median of study population of the cluster respectively). Each community variable was categorized into “low” and “high” using the national median value as the cut-off, consistent with previous DHS-based multilevel studies. Variables such as health insurance coverage, facility quality indicators, and cultural belief were excluded because they were not uniformly collected across countries. Additionally, although the World Health organizations revised its antenatal care guidelines in 2016 recommended a minimum of the eight contacts, DHS datasets across 2011–2024 uniformly capture ANC visits in terms of the four or more visits. For comparability and consistency across countries and survey year, this study excluded the updated eight-contact definition from our operationalization of community ANC utilization.

### Data management and statistical analysis

Stata version 17 was utilized for data cleaning and analysis. Prior to the analysis, the presence of the outcome variable in the DHS dataset for each country was confirmed. All the variables considered in the study were reviewed for missing values. Subsequently, the datasets from 35 SSA countries were appended and weighted to maintain sample representativeness and obtain reliable estimates and standard errors. The time to the first antenatal care (ANC) visit was analyzed using the Kaplan-Meier method, with the analysis incorporating sampling weights to account for the survey design and the total number of study participants in each country included in the analysis. Differences in survival distributions across categories of categorical variables were compared using the log-rank test.

The proportional hazards (PH) assumption was assessed using scaled Schoenfeld residuals, and the global test confirmed a violation (p = 0.0001), indicating that the Cox model would yield biased or unreliable estimates. Consequently, we employed a multivariable mixed‑effects parametric survival model assuming a Weibull distribution to account for clustering of participants within sampling units. The accelerated failure time (AFT) framework was chosen because it directly models survival time and provides an interpretable acceleration factor, quantifying how covariates shorten or lengthen the time to antenatal care initiation.

Unlike the semi‑parametric Cox model, which relies on proportional hazards, the AFT model specifies a parametric form for the baseline hazard function (e.g., Weibull, log‑logistic) and is more robust when proportionality is violated [26]. In addition, the AFT approach improves model fit and precision in large pooled datasets and yields results that are more policy‑relevant, as they can be interpreted in terms of delays in months rather than abstract hazard ratios. Furthermore, country-specific stratified analysis indicated broadly similar determinants of delayed ANC initiation across the 35 countries, supporting the validity of pooled modelling. Minor variations were observed by survey year and regional populations characteristics, consistent with descriptive differences.

In our analysis, four hierarchal models were fitted to select the model that best fits the data: null-model or model-I (a model with only sociodemographic and economic-level explanatory variables), model-II (a model with only obstetrics-level explanatory variables), and model-III (a model with community-level factors) and model-IV (a model with only potential candidate variables from sociodemographic and economic, obstetrics, and community-level factors). The " *mestreg*” command in Stata was used to fit these models. Sample weights (v005/1,000,000) were used to correct for over and under-sampling and to account for the complex survey design, enhancing the generalizability of the findings. As.

Random variability in time to ANC initiation across clusters was assessed by intra-class correlation coefficient (ICC), explained variance or proportion change in variance (PCV), and median acceleration ratio (MAR). Akaike’s information criteria (AIC), Bayesian information criteria (BIC), Log-likelihood (LL), and deviance (i.e.-2*LL) values were used for model comparison. As a result, random effects diagnostics test further confirmed that while heterogeneity exists, the overall monotonic hazard pattern was comparable across countries.

The presence of multicollinearity between explanatory variables was checked using variance inflation factor values and the values for the included variables ranged from 1.04 to 2.96, suggesting that there was no multi-collinearity. Finally, in the multivariable analysis, a p-value less than 0.05 and an adjusted acceleration factor with the corresponding 95% confidence interval was used to identify the factors associated with timely initiation of ANC among pregnant in 35 SSA countries.

### Ethical considerations

For this study, we utilized publicly available Demographic and Health Survey (DHS) data from 35 sub-Saharan African (SSA) countries. The survey procedures were approved by the ICF Institutional Review Board (IRB) and the respective host country’s IRB. As a result, no additional ethical approval was required for this analysis. However, we obtained formal permission to access the data from ICF International (referenced as AuthLetter_215093). Furthermore, the dataset accessed does not include any identifiable information about the individual participating in the study, ensuring confidentiality and privacy.

## Results

### Sociodemographic, obstetrics and community level characteristics

A weighted sample of 222,866 women was included and analyzed in the study. These women were followed retrospectively for an average of 4.05 months, with the follow-up period ranging from 1 to 10 months. Of the total sample, 62.36% (138,986) lived in rural areas, where only 37.93% (52,712) initiated ANC within the first trimester, and 40.10% (89,375) of women were from households in the poor wealth index category, with just 37.56% (33,566) starting ANC early, while women from richer households had a notably higher early initiation of ANC rate of 47.04% (41,597) (Table 2).

Among the included women, 40.63% (90,548) were from West African countries, with 44.73% (40,505) initiating ANC early. Southern African countries had the lowest proportion of ANC initiation during the first trimester, at 34.81% (14,171). Women surveyed between 2011 and 2018 had the lowest proportion of ANC initiation during the first trimester, at 37.89% (49,075), whereas those surveyed between 2019 and 2024 showed a higher proportion of timely initiation, at 46.81% (43,691). Regarding place of delivery, 18.57% (41,381) delivered at home, with only 28.61% (11,840) of these women having early ANC visits, compared to 45.85% (52,448) of women who delivered at health facilities. In terms of age distribution, 45.58% (101,587) were aged 25–49 years, with 42.94% (43,619) initiating ANC early and 39.70% (27,389) were ANC initiated among younger women aged 15–24. Regarding marital status, 72.74% (162,119) of women were married, with 41.20% (66,790) starting ANC early, while single women had a slightly higher rate of 42.76% (25,075).

Among the women included in the study, 30.99% (69,057) had no formal education, and of these, only 38.77% (26,773) initiated ANC early. A majority of women 68.10% (151,769) had access to mass media, and 44.30% (67,233) of those with media exposure were initiated ANC timely. Perceived distance to health facilities was a problem for 36.67% (75,719) of women, with only 39.33% (29,780) women initiating ANC early among this group. Additionally, 86.28% (192,284) women lived in large households, with 41.03% (78,902) initiating ANC within the first trimester. Furthermore, among the, 60.70% (135,289) of women who had a birth interval of less than 36 months, 40.44% (54,711) women were started ANC timely.

Regarding contraceptive use, 64.17% (143,012) of the women did not use any method, and only 40.11% (57,369) of them initiated ANC within the first trimester. Postnatal care utilization was low, with 63.79% (141,479) of the women not receiving postnatal care and among these, 40.68% (*n* = 57,559) women had had timely ANC visits. Women with caesarean section deliveries constituted only 7.81% (17,376), and of these, 53.94% (9,373) initiated ANC timely. Regarding parity, 22.77% (50,750) women were primiparous, and 44.87% (22,771) of them initiated ANC in the first trimester, while grand multiparous women had the lowest rate of ANC initiation timely, at 36.36% (22,671). Among the 13.96% (31,107) women who had experienced child loss, only 37.41% (11,636) women were initiated ANC timely.

A large proportion of women lived in disadvantaged communities. Specifically, 69.26% (154,367) lived in high-poverty areas, and of these, 41.51% (64,074) initiated ANC early. Similarly, 86.41% (192,581) women lived in low-education communities, and 42.03% (80,945) of these women had timely initiation of ANC. Furthermore, 35.31% (79,800) of women had low community ANC utilization and of these group, only 22.02% (17,573) initiated ANC timely, compared to 52.56% (75,193) women in high utilization communities.

**Table 2:** Sociodemographic and economic characteristic of the women and their overall background status, in 35 SSA countries, DHS from 2011 to 2024 (*n* = 222, 866)

		Time to ANC Initiation		
Variables	Categories	Event N (%)	Censored N (%)	Total weighted N (%)
Women age	15-24	27,389(39.70)	41,599(60.30)	68,988(30.96)
	25-34	43,619(42.94)	57,968(57.06)	101,587(45.58)
	35-49	21,758(41.61)	30,533(58.39)	52,291(23.46)
Women educational status	No education	26,773(38.77)	42,284(61.23)	69,057(30.99)
	Primary education	27,556(38.50)	44,027(61.50)	71,584(32.12)
	Secondary education	31,656(44.85)	38,926(55.15)	70,582(31.67)
	Higher education	6,780(58.24)	4,861(41.76)	11,642(5.22)
Place of delivery	Home	11,840(28.61)	29,541(71.39)	41,381(18.57)
	Health facility	52,448(45.85)	61,945(54.15)	114,394(51.33)
	Others	28,477(42.45)	38,613(57.55)	67,090(30.10)
Marital status	Single	25,975(42.76)	34,770(57.24)	60,746(27.26)
	Married	66,790(41.20)	95,329(58.80)	162,119(72.74)
Household size	Small	7,927(45.31)	9,568(54.69)	17,495(7.85)
	Medium	5,936(45.37)	7,149(54.63)	13,086(5.87)
	Large	78,902(41.03)	113,381(58.97)	192,284(86.28)
Mass media exposure	No	25,533(35.91)	45,563(64.09)	71,096(31.90)
	Yes	67,233(44.30)	84,536(55.70)	151,769(68.10)
Distance to health facility	Distance is problem	29,780(39.33)	45,938(60.67)	75,719(36.67)
	Distance is not problem	55,897(42.75)	74,870(57.25)	130,768(63.33)
Wealth index	Poor	33,566(37.56)	55,808(62.44)	89,375(40.10)
	Middle	17,602(39.07)	27,455(60.93)	45,057(20.22)
	Rich	41,597(47.04)	46,835(52.96)	88,432(39.68)
Place of residence	Urban	40,054(47.75)	43,826(52.25)	83,880(37.64)
	Rural	52,712(37.93)	86,273(62.07)	138,986(62.36)
Subregion	West African Countries	40,505(44.73)	50,042(55.27)	90,548(40.63)
	East African Countries	20,878(39.62)	31,817(60.38)	52,696(23.64)
	Central African Countries	17,211(44.24)	21,697(55.76)	38,908(17.46)
	Southern African Countries	14,171(34.81)	26,542(65.19)	40,713(18.27)
Survey year category	2011-2018	49,075(37.89)	80,449(62.11)	129,524(58.12)
	2019-2024	43,691(46.81)	49,651(53.19)	93,342(41.88)
Birth interval in months	< 36 months	54,711(40.44)	80,578(59.56)	135,289(60.70)
	≥ 36 months	38,055(43.45)	49,521(56.55)	87,577(39.30)
Current contraceptive using	Yes	35,397(44.33)	44,456(55.67)	79,853 (35.83)
	No	57,369(40.11)	85,643(59.89)	143,012 (64.17)
Postnatal care	Yes	34,711(43.21)	45,612(56.79)	80,323(36.21)
	No	57,559(40.68)	83,919(59.32)	141,479(63.79)
Last birth C/S	Yes	9,373(53.94)	8,002(46.06)	17,376(7.81)
	No	83,189(40.58)	121,820(59.42)	205,010(92.19)
Parity	Primipara	22,771(44.87)	27,978(55.13)	50,750(22.77)
	Multipara	47,323(43.11)	62,442(56.89)	109,765(49.25)
	Grand multipara	22,671(36.36)	39,678(63.64)	62,350(27.98)
Ever had a child loss	Yes	11,636(37.41)	19,471(62.59)	31,107 (13.96)
	No	81,130(42.31)	110,628(57.69)	191,758 (86.04)
Community ANC Utilization	Low utilization	17,573(22.02)	62,226(77.98)	79,800 (35.31)
	High utilization	75,193(52.56)	67,873(47.44)	143,066 (64.19)
Community women education	Low education	80,945(42.03)	111,636(57.97)	192,581(86.41)
	High education	11,821(39.03)	18,463(60.97)	30,284(13.59)
Community level poverty	Low poverty	28,692(41.89)	39,807(58.11)	68,499(30.74)
	High poverty	64,074(41.51)	90,292(58.49)	154,367(69.26)

### Time to first antenatal care among pregnant women in 35 sub–Saharan African countries

**Fig. 2:**
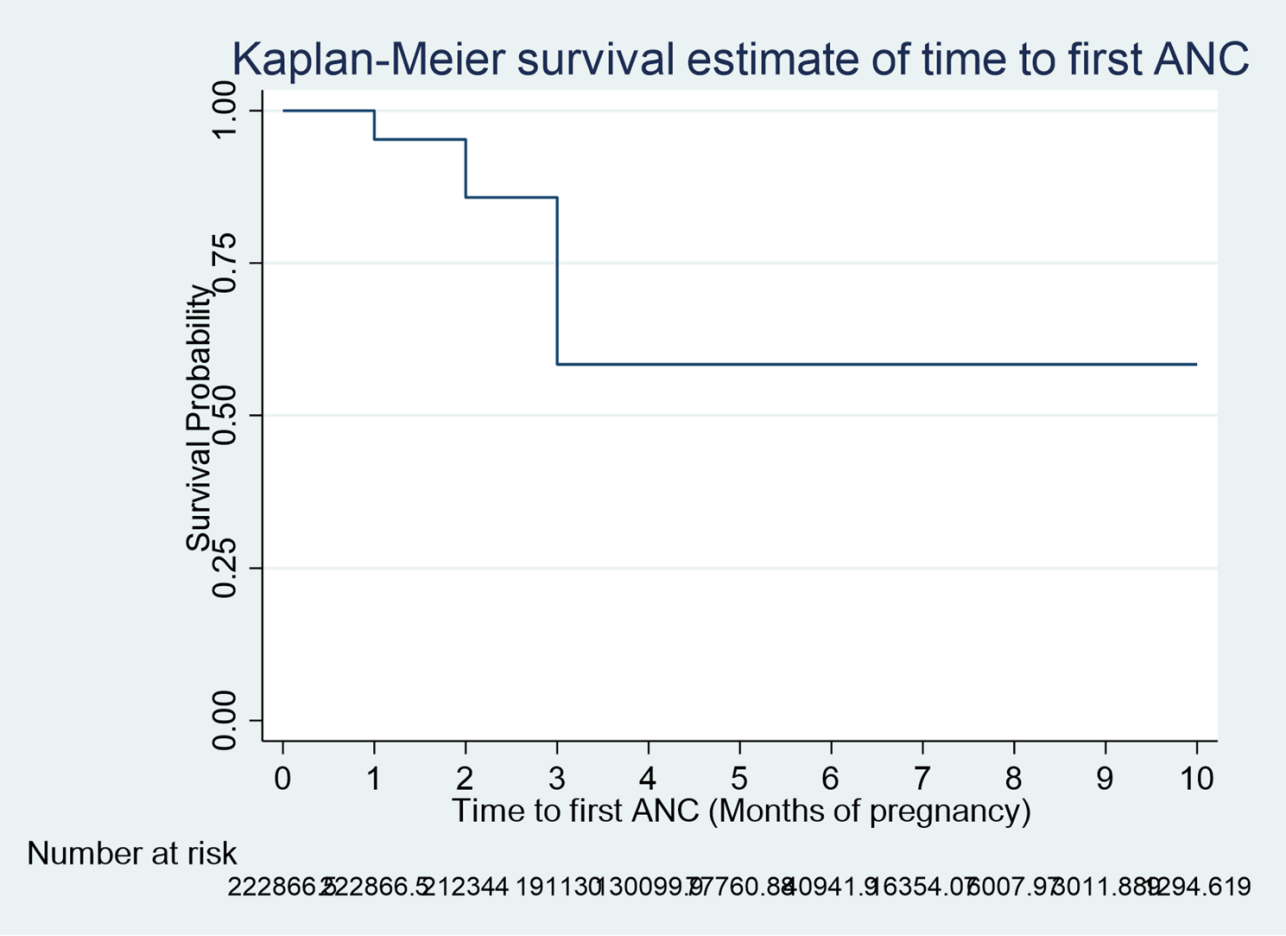
Kaplan-Meier survival curve showing the cumulative probability of pregnant women not having attended their first ANC visit, based on data from Demographic and Health Surveys (DHS) in sub-Saharan African (SSA) countries between 2011 and 2024

In this study, the timely initiation of ANC visits across 35 sub-Saharan African countries was 41.45% (95% CI: 41.25%–41.66%). The estimated mean (restricted) survival time for pregnant women to initiate their first ANC visit was 6.9 months (95% CI: 6.89–6.92). The overall incidence rate was approximately 12.8 per 100 person-months of observation. The Kaplan-Meier survival curve (Fig. 2) showed a steep drop in the survival probability between approximately months two and three, indicating that a substantial number of women initiated their first ANC visit during this period. The median time for the first antenatal care visit was 4 months (IQR: 3–5), indicating half of all pregnant women had not yet had their first ANC appointment by the time they were four months pregnant and less than half of pregnant women being booked for ANC within the first trimester.

The log-rank test result (Table 3) shows that there is statistically significant (*P*-value = 0.0001) difference in the survival experience of groups among women age, women educational status, place of delivery, household size, mass media exposure, distance to health facility, wealth index, place of residence, subregion, birth interval in months, current contraceptive using, postnatal care, last birth C/S, parity, ever had a child loss, community ANC Utilization, and community women education.

**Table 3:** Comparison of survival time of sociodemographic, obstetrics and community level factors of time to initiate ANC among women in 35 SSA countries, DHS from 2011 to 2024 (*n* = 222, 866)

		Time to ANC Initiation			
Variables	Categories	Event N (%)	Censored N (%)	Log-rank test chi-square	P-value
Women age	15-24	27,389(39.70)	41,599(60.30)	179.57	**0.0001**
	25-34	43,619(42.94)	57,968(57.06)		
	35-49	21,758(41.61)	30,533(58.39)		
Women educational status	No education	26,773(38.77)	42,284(61.23)	2117.41	**0.0001**
	Primary education	27,556(38.50)	44,027(61.50)		
	Secondary education	31,656(44.85)	38,926(55.15)		
	Higher education	6,780(58.24)	4,861(41.76)		
Place of delivery	Home	11,840(28.61)	29,541(71.39)	3822.15	**0.0001**
	Health facility	52,448(45.85)	61,945(54.15)		
	Others	28,477(42.45)	38,613(57.55)		
Marital status	Single	25,975(42.76)	34,770(57.24)	1.9	0.1684
	Married	66,790(41.20)	95,329(58.80)		
Household size	Small	7,927(45.31)	9,568(54.69)	181.1	**0.0001**
	Medium	5,936(45.37)	7,149(54.63)		
	Large	78,902(41.03)	113,381(58.97)		
Mass media exposure	No	25,533(35.91)	45,563(64.09)	1556.31	**0.0001**
	Yes	67,233(44.30)	84,536(55.70)		
Distance to health facility	Distance is problem	29,780(39.33)	45,938(60.67)	311.19	**0.0001**
	Distance is not problem	55,897(42.75)	74,870(57.25)		
Wealth index	Poor	33,566(37.56)	55,808(62.44)	2356.41	**0.0001**
	Middle	17,602(39.07)	27,455(60.93)		
	Rich	41,597(47.04)	46,835(52.96)		
Place of residence	Urban	40,054(47.75)	43,826(52.25)	1792.61	**0.0001**
	Rural	52,712(37.93)	86,273(62.07)		
Subregion	West African Countries	40,505(44.73)	50,042(55.27)	1979.73	**0.0001**
	East African Countries	20,878(39.62)	31,817(60.38)		
	Central African Countries	17,211(44.24)	21,697(55.76)		
	Southern African Countries	14,171(34.81)	26,542(65.19)		
Survey year category	2011-2018	49,075(37.89)	80,449(62.11)	1831.29	**0.0001**
	2019-2024	43,691(46.81)	49,651(53.19)		
Birth interval in months	< 36 months	54,711(40.44)	80,578(59.56)	193.21	**0.0001**
	≥ 36 months	38,055(43.45)	49,521(56.55)		
Current contraceptive using	Yes	35,397(44.33)	44,456(55.67)	344.38	**0.0001**
	No	57,369(40.11)	85,643(59.89)		
Postnatal care	Yes	34,711(43.21)	45,612(56.79)	207.07	**0.0001**
	No	57,559(40.68)	83,919(59.32)		
Last birth C/S	Yes	9,373(53.94)	8,002(46.06)	1326.56	**0.0001**
	No	83,189(40.58)	121,820(59.42)		
Parity	Primipara	22,771(44.87)	27,978(55.13)	983.45	**0.0001**
	Multipara	47,323(43.11)	62,442(56.89)		
	Grand multipara	22,671(36.36)	39,678(63.64)		
Ever had a child loss	Yes	11,636(37.41)	19,471(62.59)	192.37	**0.0001**
	No	81,130(42.31)	110,628(57.69)		
Community ANC Utilization	Low utilization	17,573(22.02)	62,226(77.98)	19504.15	**0.0001**
	High utilization	75,193(52.56)	67,873(47.44)		
Community women education	Low education	80,945(42.03)	111,636(57.97)	110.28	**0.0001**
	High education	11,821(39.03)	18,463(60.97)		
Community level poverty	Low poverty	28,692(41.89)	39,807(58.11)	0.08	0.7728
	High poverty	64,074(41.51)	90,292(58.49)		

### Result of random-effect analysis (measures of variation) and model comparison

The intraclass correlation coefficient (ICC) from the null model (Model I) revealed that approximately 3.26% of the total variation in the timing of antenatal care (ANC) initiation among pregnant women was attributable to differences between clusters, while the remaining 96.74% was explained by sociodemographic and economic-level factors. In the final model (Model IV), the proportional change in variance (PCV) reached 26%, indicating that the combined effects of sociodemographic and economic, obstetrics, and community-level factors accounted for a substantial portion of the cluster-level variation in ANC initiation.

Evidence of persistent heterogeneity across clusters was supported by the median acceleration ratio, which declined from 1.25 in the null model to 1.21 in the final model, indicating that after adjusting for covariates, women in clusters with delayed ANC initiation had approximately 21% longer median time to first ANC visit compared to those in clusters with earlier initiation. However, the reduction in the ratio (from 1.25 to 1.21) shows that the covariates explain some of the between-cluster variation. Model IV was identified as the most parsimonious and best-fitting model, as it exhibited the lowest values for Akaike’s Information Criterion (AIC = 503124.68), Bayesian Information Criterion (BIC = 503421.43), and Deviance Information Criterion (DIC = 503066.68) (see Table 4).

**Table 4:** Selection of most parsimonious model (measures of variation) at cluster or community level for timely initiation of ANC among pregnant women in 35 SSA countries, 2011–2024

Measure of variation			Models	
	Null-Model (Model I)	Model II	Model III	Model IV
Cluster-level variance	0.055	0.053	0.043	0.04
Intra-class correlation (ICC%)	3.26%	3.12%	2.6%	2.4%
Proportional change in variance (PCV%)	Reference	3.8%	20.7%	26%
Median acceleration ratio	1.25	1.24	1.22	1.21
Model summary				
Akaike’s information criteria	535521.58	580745.52	553102.36	**503124.68**
Bayesian information criteria	535716.1	580848.61	553164.25	**503421.43**
Log-likelihood	-267741.79	-290362.76	-276545.18	-**251533.34**
Deviance information criteria	535483.59	580725.52	553090.35	**503066.68**

### Determinants of timely antenatal care initiation among pregnant women in 35 sub–Saharan African Countries

Based on the mixed-effects Weibull survival regression analysis, maternal age were significant determinants of the timing of the first antenatal care (ANC) visit. Women in the younger age category (15–24 years) had an 89% longer time to initiate their first ANC visit compared to older women (35–49 years) (AAF = 0.11; 95% CI: 0.09, 0.12); and women aged 25–34 years had a 97% longer time to initiate their first ANC visit compared to older women (35–49 years) (AAF = 0.03; 95% CI: 0.02, 0.04). This indicates that older women are more likely to start their ANC earlier in pregnancy, potentially within the recommended first trimester. Similarly, the place of residence had a significant effect to the first antenatal care visit. Women residing in rural areas had a 9% longer time to start their first ANC visit than their urban counterparts (AAF = 0.91; 95% CI: 0.90, 0.93). In other words, women in rural areas were less likely to initiate ANC early in pregnancy compared to women living in urban areas (Table 5).

**Table 5:** Mixed-effects Weibull accelerated failure time survival regression analysis of determinants of time to first ANC visit among women in 35 sub-Saharan African countries, 2011–2024 (DHS)

Variables	Categories	Model I	Model II	Model III	Model IV
Maternal age category	15-24	0.03(0.02, 0.04) *			**0.11(0.09, 0.12)** **
	25-34	0.01(0.02, 0.04) *			**0.03(0.02, 0.04)** **
	35-49	**1**			**1**
Place of residence	Rural	0.09(0.08, 0.10) *			**0.91(0.90, 0.93)** **
	Urban	**1**			**1**
Women education	No education	0.30(0.28, 0.32) *			**0.95(0.92, 0.98)** **
	Primary education	0.32(0.30, 0.34) *			**0.84(0.82, 0.87)** **
	Secondary education	0.23(0.21, 0.25) *			0.83(0.80, 0.85) **
	Higher education	**1**			**1**
Place of delivery	Home	0.17(0.16, 0.18) *			0.82(0.81, 0.83) **
	Health facility	**1**			**1**
Marital status	Single	0.04(0.05, 0.014) *			**0.96(0.95, 0.98)** **
	Married	**1**			**1**
Mass media exposure	No	0.09(0.08, 0.10) *			**0.90(0.88, 0.91)** **
	Yes	**1**			**1**
Wealth index category	Poor	0.08(0.07, 0.09) *			**0.96(0.94, 0.98)** **
	Middle	0.08(0.07, 0.10) *			**0.92(0.90, 0.94)** **
	Rich	**1**			**1**
Parity	Primipara		**1**		**1**
	Multipara		0.04(0.03, 0.05) *		**0.91(0.89, 0.93)** **
	Grand multipara		0.21(0.19, 0.22) *		0.79(0.77, 0.80) **
Ever had a child loss	Yes		0.04(0.02, 0.05) *		**0.99(0.97, 1.01)**
	No		**1**		**1**
Community ANC Utilization	Low utilization			0.84(0.83, 0.85) *	0.39(0.38, 0.40) **
	High utilization			**1**	**1**

Woman’s educational attainment, the place of her last delivery, marital status, and exposure to mass media were all significant factors influencing the timing of the first antenatal care (ANC) visit. Women with no formal, primary, and secondary education had a 5%, 16%, and 17% longer time to initiate their first ANC, respectively, compared to women with higher education (AAF = 0.95, 95% CI: 0.92, 0.98; AAF = 0.84, 95% CI: 0.82, 0.87; AAF = 0.83, 95% CI: 0.80, 0.85). This suggests that women with higher levels of education are more likely to initiate the first ANC visit within the first trimester of pregnancy compared to women with no formal education.

Similarly, women whose last delivery was at home had an 18% longer time to start ANC compared to those who delivered in a health facility (AAF = 0.82; 95% CI: 0.81, 0.83), meaning they were less likely to seek care in the first trimester. Moreover, single women experienced a 4% longer time to initiation ANC at first trimester of pregnancy than married women (AAF = 0.96; 95% CI: 0.95, 0.98). Furthermore, women not exposed to mass media had a 10% longer time to start their first ANC visit within first trimester than those with exposure (AAF = 0.90; 95% CI: 0.88, 0.91), suggesting women who had exposed to mass media are more likely to initiate first trimester ANC.

Women in the poor and middle wealth index categories had a 4% and 8% longer time, respectively, to initiate their first ANC visit compared to women in the rich wealth index category (Poor: AAF = 0.96; 95% CI: 0.94, 0.98; Middle: AAF = 0.92; 95% CI: 0.90, 0.94). This indicates that poorer women are less likely to start ANC early in the first trimester of pregnancy than wealthier women. Additionally, multiparous women had a 9% longer time (AAF = 0.91; 95% CI: 0.89, 0.93) and grand multiparous women had a 21% longer time (AAF = 0.79; 95% CI: 0.77, 0.80) to start their first ANC visit compared to primiparous women. In other words, women with more children were less likely to initiate their first ANC visit within the first trimester of pregnancy than women having their first child.

Furthermore, this study revealed that community-level factors were significant determinants of the timing of the first antenatal care (ANC) visit. Women residing in communities characterized by low ANC utilization had a 61% longer time to start their first ANC visit compared to those in high-utilization communities (AAF = 0.39; 95% CI: 0.38, 0.40). This indicates that women in areas where ANC service uptake is generally low are less likely to initiate their first ANC visit within the first trimester of pregnancy.

## Discussion

Timely initiation of antenatal care (ANC) is a major challenge in sub-Saharan Africa, with only 41.5% (95% CI: 41.25%–41.66%) of pregnant women starting care within the recommended first trimester. The mean time to the first ANC visit was 6.9 months (95% CI: 6.89, 6.92), indicating pregnant women in this population remained without any ANC care for nearly 7 months. The median time for the first antenatal care visit was 4 months (IQR: 3–5), indicating half of all pregnant women had not yet had their first ANC appointment by the time they were four months pregnant and less than half of pregnant women being booked for ANC within the first trimester.

This study identified key determinants at the sociodemographic and economic, obstetrics and community levels factors. Significant factors associated with delayed ANC initiation included younger maternal age, rural residence, lower educational attainment, and delivering a previous last child at home. Furthermore, being single, lacking mass media exposure, poor household wealth, and higher parity (having more children) were also associated to delay. Moreover, at the community level, residing in an area with low overall ANC utilization was a strong predictor of late initiation of ANC in the first trimester.

The mean survival time to the first ANC visit was 6.9 months (95% CI: 6.89, 6.92), indicating pregnant women in this population remained without any ANC care for nearly 7 months. The finding is higher than previous study conducted in Ethiopia(6.8 months) [5]. It also higher than the mean of 3.4 gestational months reported in research from Ghana [29].

The median time for the first antenatal care visit was 4 months (IQR: 3–5), indicating half of all pregnant women had not yet had their first ANC appointment by the time they were four months pregnant and less than half of pregnant women being booked for ANC within the first trimester. The finding is in line with study conducted in Ethiopia (4 months) [30, 31], and India (4 months) [32]. However, the finding is lower than prior study conducted in Ethiopian data, which indicated a median survival time of 7 months [33], Ethiopia(5 months) [28, 34, 35], Tanzania(5 months) [36], Nigeria (6 months) [37], and Uganda (7 month) [38] and, underscoring a trend of later initiation within the national context.

The prevalence of timely antenatal care (ANC) initiation within the first trimester of pregnancy was found to be 41.45% (95% CI: 41.25%–41.66%). The finding is significantly higher than studies conducted in Afghanistan (33.1%) [39], Tanzania(31.2%) [40], Benin(24.6%) [41], Kenya(38.2%) [42], Ethiopia(38%) [25], systematic review and meta-analysis in Ethiopia(37%) [43], and Africa(37.5%) [44]. However, the finding is lower than previous studies conducted in Ethiopia (42.7%) [5], Ethiopia(62.64%) [28], Pakistan(48%) [45], Ghana(57%) [29], 54 low- and middle-income countries, with 69.1% in Central and Southern Asia, 63.5% in Eastern and Southeast Asia, 68.1% in Latin America, and the Caribbean and 54.6% in Northern America and Western Europe [46], South Asia (59.5%) [47], India (69.3%) [48], Bangladesh (43.0%) [49], and Vietnam (75.2%) [50]. The possible discrepancy might be arisen from variation in methodology, healthcare context across countries, health policy implementation, different study period, widespread education, and economically developed regions [51].

Women in the younger age categories (15–24 years and 25–34 years) had an 89% and 97% longer time, respectively, to initiate their first ANC visit compared to older women (35–49 years). This indicates that older women are more likely to start their ANC earlier in pregnancy, potentially within the recommended first trimester. This finding is in line with previous studies India [52], Ethiopia [53], Nepal [54], and Ghana [55]. The possible explanation for this association could be that younger women, particularly adolescents, often face unique barriers to accessing healthcare, including lack of autonomy, fear of stigma, financial dependence, and lower health literacy regarding the importance of early antenatal care (ANC) initiation [56].

Women residing in rural areas had a 9% longer time to start their first ANC visit than their urban counterparts. In other words, women in rural areas were less likely to initiate ANC early in pregnancy compared to women living in urban areas. The finding is similar with previous study conducted in Ethiopia [5, 28, 57], India [32], sub-Saharan Africa [25], Nigeria [37], and Kenya [58]. The possible justification for this could be that urban women may have an advantage in seeking healthcare because they have better access to transportation and social media, which provides them with information about facility services, timeliness, and importance [5]. Additional reason could be that women in rural settings frequently have lower levels of education and income, which correlates with lower health literacy and a diminished perception of the importance of early prenatal visits, delaying their engagement with the health system [59].

Women with no formal, primary, and secondary education had a 5%, 16%, and 17% longer time to initiate their first ANC, respectively, compared to women with higher education. This suggests that women with higher levels of education are more likely to initiate the first ANC visit within the first trimester of pregnancy compared to women with no formal education. The finding is similar with previous study conducted in Ethiopia [5, 28], India [32], Nepal [60], A multicounty analysis of DHS in SSA [25], and Nigeria [37]. The possible justification could be that women with higher education are more likely to be employed and financially independent, and are better informed about the health necessities of pregnancy [5]. This awareness and autonomy enable them to recognize the critical importance of scheduling their first ANC visit early [61].

Women whose last delivery was at home had an 18% longer time to start ANC compared to those who delivered in a health facility, meaning they were less likely to seek ANC care in the first trimester. This finding is similar with study conducted in Ethiopia [5, 62] and Kenya [42]. The possible justification could be exposure to health education during institutional delivery is a potential mechanism for improving maternal attitudes, as the advice received likely emphasizes the critical importance of early antenatal booking for maternal and fetal health [5].

Women not exposed to mass media had a 10% longer time to start their first ANC visit within first trimester than those with exposure, suggesting women who had exposed to mass media are more likely to initiate first trimester ANC. The finding is agreed with previous study conducted in Ethiopia [28, 34, 63], and India [64]. The possible explanation could be that media access is a key indicator of broader socioeconomic advantage, including higher wealth, urban residency, educational attainment, and healthcare availability [28, 65]. These intersecting factors enhance women’s autonomy, enabling them to utilize healthcare services more effectively. This empowerment is critical for improving maternal and fetal health outcomes, particularly through the timely initiation of antenatal care (ANC) [66].

Single women experienced a 4% longer time to initiation ANC at first trimester of pregnancy than married women. This indicates that the partner’s presence during pregnancy is associated with a reduced time to first antenatal care visit, emphasizing the critical role of male involvement in maternal healthcare and family planning. The finding is similar with previous study conducted in Ethiopia [5], South Africa [67], and Rwanda [14]. This might be due to the fact that the fear of social stigma can undermine a woman’s confidence in seeking care when the paternity of her child is uncertain or unacknowledged [67]. Additionally, the burden of solely managing the responsibilities of pregnancy and preparing for the child’s life may be a significant barrier and late recognition or acceptance of the pregnancy common in cases of unintended or mistimed pregnancies often results in delayed ANC initiation, as women may be disengaged from the process early on [68].

Women in the poor and middle wealth index categories had a 4% and 8% longer time, respectively, to initiate their first ANC visit compared to women in the rich wealth index category. This indicates that poorer women are less likely to start ANC early in the first trimester of pregnancy than wealthier women. This finding is similar with previous study conducted in Ethiopia [28, 30–70], and Pakistan [45]. The possible justification could be that women from higher-income households benefit from greater autonomy, advanced education, and increased self-efficacy, facilitating their access to services [71–73]. Conversely, women from impoverished backgrounds encounter prohibitive barriers, including familial obligations that restrict their mobility and hidden indirect costs such as transportation [30].

Multiparous women had a 9% longer time and grand multiparous women had a 21% longer time to start their first ANC visit compared to primiparous women. In other words, women with more children were less likely to initiate their first ANC visit within the first trimester of pregnancy than women having their first child. The finding is supported with previous study conducted in Ethiopia [5, 28, 69], India [32], Kenya [68], Nigeria and Malawi [74]. This justification could be attributed to two contrasting factors among multiparous women: heightened self-assurance from previous pregnancies, which may reduce perceived urgency, and conversely, negative prior experiences with the health system or logistical constraints such as time and resource limitations that hinder early initiation of ANC [75].

Women residing in communities characterized by low ANC utilization had a 61% longer time to start their first ANC visit compared to those in high-utilization communities. This indicates that women in areas where ANC service uptake is generally low are less likely to initiate their first ANC visit within the first trimester of pregnancy. This might be due to the fact that communities with low ANC utilization are often also areas of concentrated poverty and lower average educational attainment [76]. Additionally, individual behavior is heavily influenced by the perceived behavior of others, known as descriptive norms. In communities where late initiation or low ANC use is common, it becomes the socially accepted norm [77].

### Strength and limitation of the study

This study utilized on a recent, nationally representative dataset from 35 sub‑Saharan African countries collected between 2011 and 2024, providing a substantial sample size that ensures high statistical power and strengthens the reliability of the findings. The inclusion of diverse populations across the region enhances the generalizability of results to pregnant women in sub‑Saharan Africa. A major methodological strength is the use of a multilevel mixed‑effects acceleration failure time model, which appropriately accounts for the hierarchical structure of the data (women nested within communities and countries) and efficiently manages right‑censored observations common in time‑to‑event analyses. Together, these features provide robust evidence for identifying predictors of timely initiation of antenatal care.

However, important health system variables including health insurance coverage, the number and type of facilities, the standard of ANC services, women’s perceptions of care, and broader country‑level factors such as political stability were not assessed, representing additional limitations of this study. Additionally, the cross‑sectional design of the survey prevents the establishment of causal relationships between independent and dependent variables, restricting interpretation to associations rather than definitive causal inferences. The study did not directly correlate initiation timing with pregnancy outcomes such as maternal morbidity, neonatal mortality, or birth weight. A further limitation is that our measure of community ANC utilization was based on the earlier WHO Focused Antenatal Care model (≥ 4 visits), rather than the updated 2016 recommendation of eight contacts. This discrepancy reflects the structure of DHS data rather than a conceptual oversight, but it may underestimate the gap between current global standards and actual practice in sub-Saharan Africa.

## Conclusion

In conclusion, this large-scale retrospective analysis pooling DHS data from 35 Sub-Saharan African countries (2011–2024) and applying a multilevel mixed-effects Weibull accelerated failure time model revealed that, timely initiation of antenatal care (ANC) remains a major challenge in 35 sub‑Saharan African countries, with only 41.5% of pregnant women starting care within the recommended first trimester according to the World Health Organization. The mean time to the first ANC visit was 6.9 months, meaning many women remained without essential maternal health services for nearly seven months. The median time of four months further indicates that half of all pregnant women had not yet received ANC by the end of the first trimester.

This delay has serious implications for maternal health service delivery and newborn health, as it increases the risk of undetected complications and missed opportunities for preventive interventions. The findings highlight that younger maternal age, rural residence, low educational attainment, poverty (poor and middle household wealth), being single, higher parity, home delivery, lack of media exposure, and low community ANC utilization are key determinants that must be addressed to improve maternal health outcomes and reduce preventable mortality.

To respond to these challenges, targeted interventions should prioritize the integration of timely ANC initiation into national maternal health strategies. Ministries of Health should expand access to adolescent-friendly services, and mobile clinics as well policy integration, and resource allocation, while local governments oversee implementation, provide financial incentives, transport subsidies to reduce geographic and economic barriers. National media authorities and community organizations should collaborate to deliver culturally tailored education campaigns that normalize first-trimester ANC initiation. International partners and NGOs can support capacity building by training health workers, strengthening service quality, and ensuring adequate supply chains for maternal health services. Together, these multi-level actors must coordinate efforts to prioritize timely ANC initiation as a cornerstone of maternal health policy, thereby reducing preventable maternal and neonatal morbidity and mortality across Sub-Saharan Africa.

Additionally, community-based education campaigns should be designed by peer-led group discussion facilitated by community health workers, school-based campaigns that integrate ANC awareness in to secondary school health curricula that can reach adolescent before pregnancy, and engagement of religious leaders, traditional birth attendants and traditional elders are critical. Addressing these barriers requires a coordinated, multi‑level strategy that prioritizes timely ANC initiation as a cornerstone of maternal health policy in sub‑Saharan Africa.

Future studies should incorporate health system variables such as facility availability, service quality, health insurance coverage and women’s perceptions of ANC, alongside country‑level factors like political stability. Longitudinal research designs are recommended to establish causal relationships and guide more effective policy and intervention strategies, as they reduce recall bias, provide richer causal evidence for improving timely ANC uptake in Sub-Saharan Africa, and allow researchers to follow women prospectively to ensure that exposures such as education, parity, or community poverty precede the outcome of ANC initiation. Future research should explicitly correlate ANC initiation timing with pregnancy outcomes to provide stronger evidence for policy prioritizations. Furthermore, future DHS surveys and regional analyses should adopt the revised WHO definition of eight antenatal contacts to ensure alignment with global maternal health standards.

## Supplementary Information

Supplementary Material 1.

## Data Availability

The DHS datasets analyzed during the current study are publicly available from the DHS Program website ( [https://dhsprogram.com/data/](https:/dhsprogram.com/data) ).

## Abbreviations

AIC: Akaike’s Information Criteria
AAF: Adjusted Acceleration Failure
ANC: Antenatal Care
BIC: Bayesian Information Criteria
CI: Confidence Interval
DHS: Demographic and Health Survey
DIC: Deviance Information Criterion
EAs: Enumeration Areas
IQR: Inter-Quartile Range
ICC: Intra-class Correlation Coefficient
IRB: Institutional Review Board
LL: Log-Likelihood
MAR: Median Acceleration Ratio
PCV: Proportional Change in Variance
SSA: Sub-Saharan Africa
WHO: World Health Organization
